# Immunogenicity and Efficacy of Zika Virus Envelope Domain III in DNA, Protein, and ChAdOx1 Adenoviral-Vectored Vaccines

**DOI:** 10.3390/vaccines8020307

**Published:** 2020-06-16

**Authors:** César López-Camacho, Giuditta De Lorenzo, Jose Luis Slon-Campos, Stuart Dowall, Peter Abbink, Rafael A. Larocca, Young Chan Kim, Monica Poggianella, Victoria Graham, Stephen Findlay-Wilson, Emma Rayner, Jennifer Carmichael, Wanwisa Dejnirattisai, Michael Boyd, Roger Hewson, Juthathip Mongkolsapaya, Gavin R. Screaton, Dan H. Barouch, Oscar R. Burrone, Arvind H. Patel, Arturo Reyes-Sandoval

**Affiliations:** 1 The Jenner Institute, Nuffield Department of Medicine, University of Oxford, The Henry Wellcome Building for Molecular Physiology, Roosevelt Drive, Oxford OX3 7BN, UK; cesarlc@well.ox.ac.uk (C.L.-C.); young.kim@some.ox.ac.uk (Y.C.K.); 2 Wellcome Centre for Human Genetics, Nuffield Department of Medicine, University of Oxford, Oxford OX3 7BN, UK; jmongkol@well.ox.ac.uk (J.M.); gavin.screaton@medsci.ox.ac.uk (G.R.S.); 3 MRC-University of Glasgow Centre for Virus Research, University of Glasgow, Glasgow G61 1QH, UK; Giuditta.DeLorenzo@glasgow.ac.uk (G.D.L.); j.doig@ed.ac.uk (J.C.); dwanwisa@well.ox.ac.uk (W.D.); Arvind.Patel@glasgow.ac.uk (A.H.P.); 4 Molecular Immunology Group, International Centre for Genetic Engineering and Biotechnology, Padriciano 99, 34149 Trieste, Italy; joseslon@well.ox.ac.uk (J.L.S.-C.); poggiane@icgeb.org (M.P.); burrone@icgeb.org (O.R.B.); 5 Public Health England, National Infection Service, Porton Down, Salisbury SP4 0JG, UK; stuart.dowall@phe.gov.uk (S.D.); Victoria.Graham@phe.gov.uk (V.G.); steviewils@hotmail.com (S.F.-W.); emma.rayner@phe.gov.uk (E.R.); roger.hewson@phe.gov.uk (R.H.); 6 Center for Virology and Vaccine Research, Beth Israel Deaconess Medical Center, Harvard Medical School, Boston, MA 02215, USA; pabbink@bidmc.harvard.edu (P.A.); larocca.rafael@gmail.com (R.A.L.); mjboyd100@gmail.com (M.B.); dbarouch@bidmc.harvard.edu (D.H.B.); 7 Dengue Haemorrhagic Fever Research Unit, Office for Research and Development, Faculty of Medicine Siriraj Hospital, Mahidol University, Bangkok 10700, Thailand; 8 Division of Medical Sciences, John Radcliffe Hospital, University of Oxford, Oxford OX3 9DU, UK

**Keywords:** ZIKV, DENV, EDIII, vaccine, adenovirus

## Abstract

The flavivirus envelope protein domain III (EDIII) was an effective immunogen against dengue virus (DENV) and other related flaviviruses. Whether this can be applied to the Zika virus (ZIKV) vaccinology remains an open question. Here, we tested the efficacy of ZIKV-EDIII against ZIKV infection, using several vaccine platforms that present the antigen in various ways. We provide data demonstrating that mice vaccinated with a ZIKV-EDIII as DNA or protein-based vaccines failed to raise fully neutralizing antibodies and did not control viremia, following a ZIKV challenge, despite eliciting robust antibody responses. Furthermore, we showed that ZIKV-EDIII encoded in replication-deficient Chimpanzee adenovirus (ChAdOx1-EDIII) elicited anti-ZIKV envelope antibodies in vaccinated mice but also provided limited protection against ZIKV in two physiologically different mouse challenge models. Taken together, our data indicate that contrary to what was shown for other flaviviruses like the dengue virus, which has close similarities with ZIKV-EDIII, this antigen might not be a suitable vaccine candidate for the correct induction of protective immune responses against ZIKV.

## 1. Introduction

The Flavivirus genus includes a broad range of pathogenic viruses, some of which are transmitted by the bite of infected hematophagous arthropods [1]. These viruses, including dengue (DENV), yellow fever (YFV), and West Nile (WNV) among others, are the causal agents of a wide variety of conditions that include mild, severe, and fatal hemorrhagic and neurological diseases [2,3,4]. Until recently, the Zika virus (ZIKV) was a relatively unknown member of the group, and before the Pacific Island epidemics in 2013 and 2014, ZIKV infections were mostly mild and sporadically reported in Africa [5]. Following the 2015 epidemic in Brazil, ZIKV reached a global distribution, geographically overlapping with DENV, and is now associated with neurotropic disease [6] and congenital Zika syndrome [7].

Like other flaviviruses, ZIKV is a single-stranded, positive-sense RNA virus with a host-derived double-layered lipid envelope. Its genome encodes a single viral polyprotein that is co- and post-translationally processed into 10 mature viral proteins [8]. The capsid (C), pre-membrane (prM), and envelope (E) proteins are structurally required to form a viral particle; while NS1, NS2a, NS2b, NS3, NS4a, NS4b, and NS5, perform non-structural functions, such as polypeptide post-translational processing and RNA replication. The viral surface is covered by 180 copies of E, which are arranged in 90 antiparallel dimers and distributed in a herringbone configuration [9,10]. Each copy of the E protein folds into a rod-like structure and is composed of three structural domains—domains I and II (EDIDII) form an elongated finger-like structure that expands distally into a glycine-rich, highly-hydrophobic fusion loop (FLE), which is conserved in all flaviviruses and serves a fundamental role in initiating infection [11]. Contrary to EDIDII, the Ig-like domain III (EDIII) is highly variable and has been described as the putative site for host cell-receptor binding [12].

Similar to other flavivirus infections, the E protein is the main target of the antibody response against ZIKV [13,14,15]. Although neutralizing epitopes have been identified in all three E domains for several flaviviruses [16,17,18,19], the immune response following infection is heavily dominated by anti-EDIDII antibodies, most of which target the FLE and tend to be poorly neutralizing and highly cross-reactive [20,21]. Conversely, due to the high variability and function of the domain, anti-EDIII antibodies are usually highly specific and strongly neutralizing [22,23]. Based on these features, EDIII has been used in several vaccine platforms [24], including recombinant protein-based vaccines [25], virus-like particles (VLPs) vaccines [26], and genetic vaccines such as DNA- [27,28,29] and adenovirus-based vaccines [30]. Due to the close structural and biological similarity among flaviviruses, ZIKV EDIII is a highly attractive target for pre-clinical vaccine developments.

In this study, we tested ZIKV EDIII-based vaccines candidates using different immunization platforms, including DNA-, viral-vectored (adenovirus), and recombinant protein-based approaches in mice. Our data suggest that, contrary to other flaviviruses, the polyclonal anti-EDIII response is not sufficient to confer protection in the context of ZIKV infection. These results open up an exciting new avenue for the further exploration of ZIKV EDIII antigenic features in ZIKV biology and vaccinology.

## 2. Results

### 2.1. EDIII-CH3 DNA-Based Immunization Induces Poorly Neutralizing Antibody Responses

**Figure 1:**
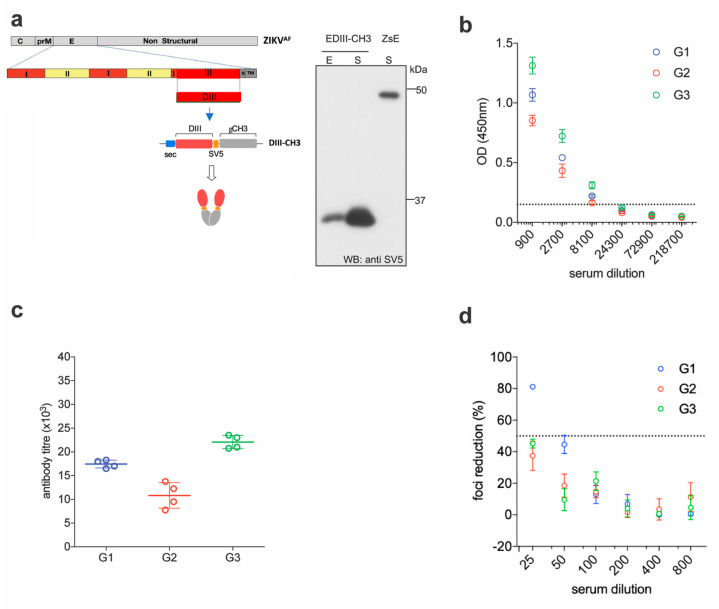
Functional immunogenicity of a DNA vaccine expressing ZIKV EDIII. (**a**) Schematic representation of constructs encoding ZIKV EDIII fused to the dimerizing γCH3 domain, based on an African strain of the Zika virus (ZIKA^AF^) (left) and Western blot (anti-SV5) of cellular extracts (E) and supernatants (S) from HEK293T cells transfected with the indicated constructs. Soluble ZIKV E protein from the supernatant of cells transfected with a plasmid construct expressing the SV5-tagged protein is shown for size comparison. (**b**) Pooled sera from DNA immunized mice were tested for ELISA reactivity on ZDIII-coated plates; the dashed line marks the detection limit of the assay. (**c**) Antibody titers from the curves shown in (b). (**d**) Foci reduction neutralization test (FRNT) of ZIKV on Vero cells with pooled sera from mice immunized with ZDIII-CH3.

We previously showed that an antigenic design comprised of the EDIII domain from all four DENV serotypes, C-terminally fused to the dimerizing constant domain 3 of the human IgG heavy chain (γCH3), not only promotes efficient secretion of properly-folded EDIII from transfected mammalian cells, but also induces robust long-term virus-specific neutralizing antibody responses in mice, when administered as a gene gun-mediated DNA vaccine [28]. Here, we decided to build on that concept and apply it to develop a DNA and a protein-based vaccine against ZIKV. The EDIII sequence from the reference ZIKV African strain MR-766 (amino acids 300–421 of the full E protein) was initially selected due to the availability of the homologous ZIKV strain, to perform functional assays. This strain has gone through multiple passages in mice, and maintains virulence in this animal model [31,32]. Codon optimized for mammalian expression, was cloned into a pVax expression vector between a human Ig-derived secretion leader peptide (sec) [33] and the dimerizing γCH3 domain. The SV5 tag (GKPIPNPLLGLD) was also included to facilitate detection and purification of the fusion protein (EDIII-CH3) (Figure 1a, left panel). Anti-SV5 tag antibodies could be induced upon vaccination but these were not assessed as they were not part of the ZIKV DIII antigen. Since antigen availability is of paramount importance in genetic immunizations [28,34], expression and secretion of the protein was tested in transiently transfected HEK293T cells, which revealed a highly efficient secretory phenotype (Figure 1a, right panel).

For the DNA immunizations, three groups of six female BALB/c mice were immunized by intradermal gene gun delivery of the plasmid DNA encoding the EDIII-CH3 protein. Each mouse received three doses of DNA (1 µg dose/animal) at 15 days intervals; sera were collected two weeks after completion of the protocol and pooled for analysis. Sera dilutions showed that all mice developed anti-ZIKV E antibody responses that were able to bind ZIKV E in ELISA (Figure 1b). As expected, anti-EDIII titers were detected in all three groups of immunized animals with mean titers ranging from 1 × 10^4^ to 2.25 × 10^4^ (Figure 1c). We then used the Foci Reduction Neutralization test (FRNT) to measure the neutralizing activity of the sera in vitro. Surprisingly, although all groups showed some degree of neutralizing activity, none reached 100% neutralization of ZIKV, and the FRNT50 titers were detected only in one group (Figure 1d). These results indicate that, despite being able to drive a robust anti-EDIII response, the anti-EDIII antibodies induced by DNA-immunization showed a low ZIKV neutralizing activity.

### 2.2. EDIII-CH3 Protein-Based Immunization Induce Poorly Neutralzsing Antibody Responses

**Figure 2:**
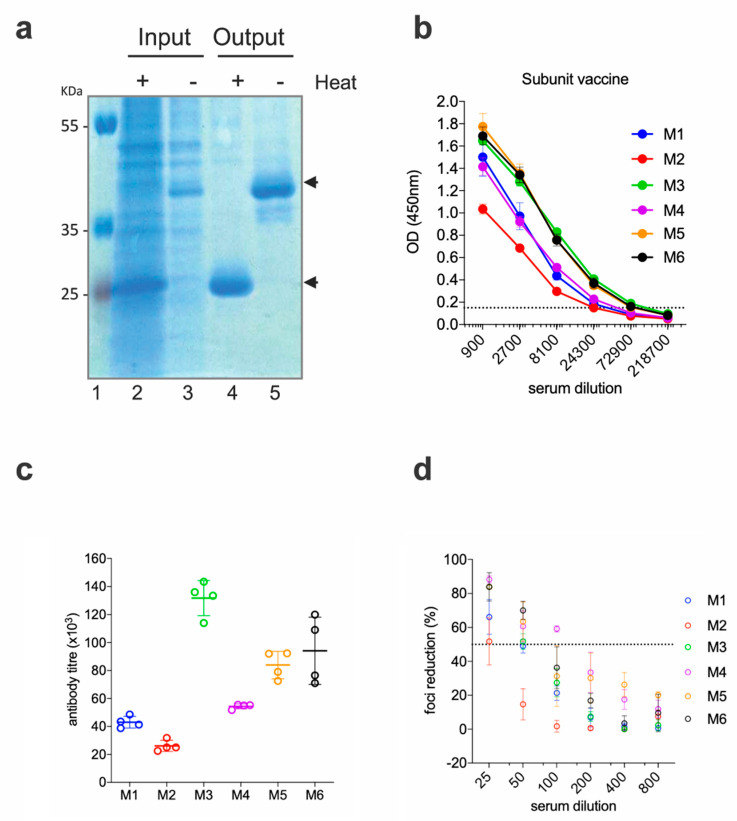
Functional immunogenicity of a subunit vaccine expressing ZIKV EDIII (**a**) SDS-PAGE of the purified protein. Cell supernatant (Input) and post-purification (Output) samples were analyzed in SDS-PAGE, following heat treatment (or not). Black arrows show the position of the monomeric (+heat) and dimeric (−heat) EDIII-CH3. (**b**) Sera from immunized mice were tested for ELISA reactivity on ZEDIII-coated plates; the dashed line marks the limit of the assay. (**c**) Antibody titers from the curves shown in (b). (**d**) Foci reduction neutralization test (FRNT) of ZIKV on Vero cells with sera from mice immunized with ZEDIII-CH3.

To rule out that the low neutralizing activities of the anti-EDIII responses were due to a factor inherent to the DNA immunization technique (i.e., altered in vivo production of the antigen, low levels of protein availability, etc.), we produced and purified the EDIII-CH3 antigen and administered it as a protein-based vaccine. EDIII-CH3 was purified from the supernatant of transfected Expi293F cells with anti-SV5 agarose affinity gel; as expected from its design, the protein was obtained as a non-covalent dimer that is disassociated upon heat treatment (Figure 2a).

Six BALB/c mice were immunized with 10 µg of the EDIII-CH3 protein using 1% ALUM MPLA (monophosphoryl lipid A) as adjuvant. Each mouse received, subcutaneously, three doses of the protein at 15 days intervals, and sera were collected two weeks after the third dose. As shown in Figure 2b, all mice developed strong anti-EDIII responses, which were higher in titer when compared to the gene-gun immunized mice (Figure 2c). As a result of the higher antibody titer and in contrast to results obtained for the DNA vaccine, FRNT50 titers were detected in all six mice; although, as before, none reached 100% neutralization of the virus (Figure 2d).

**Figure 3:**
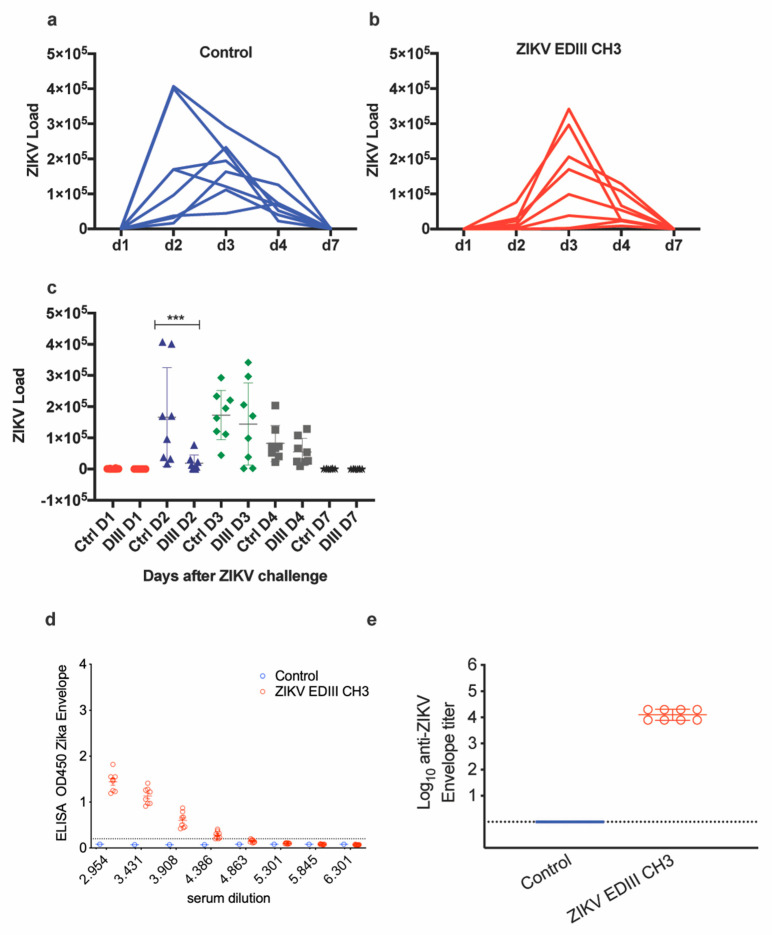
Assessment of protective efficacy induced by protein-based EDIII vaccine. Naïve (**a**) and vaccinated (**b**) mice (*n* = 8) were intravenously challenged at four weeks after vaccination with 10^4^ PFU of ZIKV- PRVABC59 strain. Upon ZIKV challenge, the viral load was monitored for up to seven days. Graphs show days post-challenge on the *x*-axis versus viral load on the *y*-axis. Continuous blue and red lines represent one mouse each for each of the control and vaccinated groups. (**c**) Peaks of viral titers for each individual mouse and for each group. (**d**) Sera from immunized mice were tested for ELISA reactivity on ZDIII-coated plates; the dashed line marks the limit of the assay. (**e**) Antibody titers from the curves shown in (b). *** *p* < 0.001

The protective efficacy of the EDIII-CH3 protein-based vaccines was further assessed in Interferon Type-I Receptor knockout mice, A129, which are highly susceptible to the ZIKV infection [35]. In this case, 8 mice were immunized with the EDIII-CH3 protein, following the same protocol and were challenged with 10^4^ PFU ZIKV PRVABC59 strain. Availability of an Asian-origin virus permitted the efficacy assessment of a heterologous challenge in mice vaccinated with an African lineage antigen. Similar to the control group (Figure 3a), protein-immunized mice were not able to contain ZIKV infection, as demonstrated by the increasing viral load titers detected in all mice after the challenge (Figure 3b), even though the peak of the viral titers was detected at a later time (Figure 3c). An analysis of the area under the curve (AUC) did not show significant differences (218,197 with 95% CI 23,273 to 413,121 for EDIII-CH3 vs. 421,938 with 95% CI 164,150 to 679,727 for the control, *p* = 0.2344) Failure to prevent viral infection was not related to a diminished response to the EDIII-CH3 antigen, as all pre-challenge sera showed consistent anti-EDIII antibody responses (Figure 3d) with a mean reciprocal titer of >4 (Figure 3e).

### 2.3. Adenoviral Vaccine Design Carrying ZIKV EDIII

**Figure 4:**
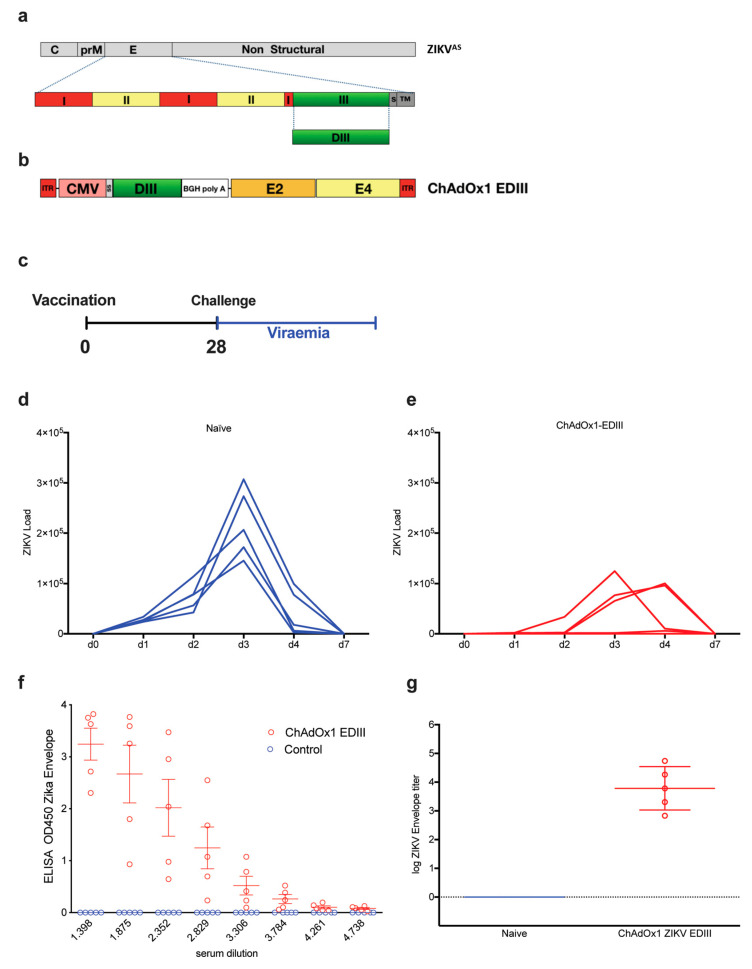
Assessment of protective efficacy induced by the ChadOx1-EDIII vaccine. (**a**) Schematic representation of the ZIKV^AS^ genome in gray, designed from an Asian lineage (ZIKA^AS^). Bottom row represents a magnified schematic of the envelope of ZIKV, with domains I, II, and III, shown in different colors. DIII is shown in green. The EDIII coding region was used to produce the recombinant adenoviral vector, containing the ZIKV EDIII (**b**). (**c**) Vaccination strategy and timeline for a challenge with a ZIKV of a homologous Asian lineage [37]. Naïve (**d**) and vaccinated (**e**) BALB/c mice (*n* = 5) were intravenously challenged with 100 PFU of ZIKV-BR strain. Upon ZIKV challenge, viral loads were monitored for up to seven days. Graphs show days post-challenge on the x-axis versus viral loads on the y-axis. Continuous blue and red lines represent one mouse each, for each of the control (d) and vaccinated groups (e). (**f**) ELISAS from the control and vaccinated groups, OD450 were recorded from 3-fold dilutions. (**g**) Log of endpoint titers from ELISA are shown.

To determine if the poorly neutralizing responses obtained with the DNA and protein-base EDIII-CH3 vaccines were due to a problem of antigenic design or rather due to an incapacity of the anti-EDIII polyclonal response to neutralize ZIKV effectively, we next evaluated a replication deficient chimpanzee adenoviral vector (ChAdOx1) as an immunization platform. Based on a previously described prME ΔTM-encoding adenoviral vectored- ZIKV vaccine [36], we constructed a ChAdOX1 encoding a codon-optimized ZIKV EDIII sequence (Figure 4a) cloned between the tPA signal sequence and a transcription termination sequence (Figure 4b). We created a consensus sequence from Asian lineages (ZIKV^AS^) to maintain consistency with a previous publication, whilst expanding our observations beyond the African strain-based designs that are dimeric, to a monomeric antigen based on Asian lineages. We then tested the ability of the ChAdOx1-EDIII vaccine to protect BALB/c mice upon an intravenous ZIKV challenge of 100 PFU, four weeks after a single immunization (1 × 10^8^ IU/mouse), using an Asian-lineage of ZIKV^AS^, ZKV2015 (Figure 4c). ZIKV challenge in naïve mice (*n* = 5) displayed a typical onset of viremia after challenge, with a peak by day 3 (Figure 4d) and the clearance of viral load in blood by day 7. Similar results to that of the EDIII protein vaccine were observed, as ZIKV replication was detected in both vaccinated and control mice, albeit all mice immunized with ChAdOx1-EDIII showed signs of protection as viral loads were significantly lower than those obtained in the control group (Figure 4e). An analysis of area under the curve (AUC) indicated that the vaccinated group displayed significantly better protection than the mock vaccinated group (105,567 with 95% CI 1,425-209,709 for EDIII vs 363,416 with 95% CI 244,574 to 482,257 for the control, *p* = 0.0126). Interestingly, a delay of viral peak at day 4 was observed in 2 out of 5 mice and complete protection with absence of viremia was observed in one mouse. Assessment of anti-EDIII antibodies in the pre-challenge sera, revealed high titers of anti-ZIKV E antibodies in all the animals vaccinated with ChAdOx1-EDIII (Figure 4f,g). Overall, the data obtained from the EDIII-CH3 DNA, subunit and viral vectored vaccines suggest that ZIKV EDIII confers suboptimal protection upon a ZIKV challenge.

### 2.4. Immunogenicity and Efficacy of ChAdOx1-EDIII in A129 Mice

**Figure 5:**
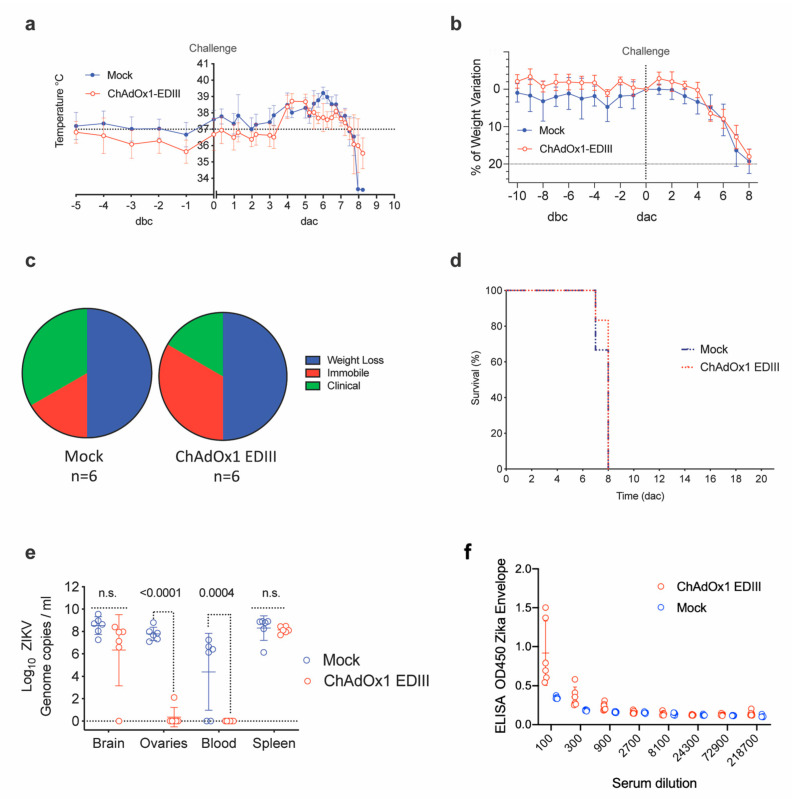
Efficacy parameters after a ZIKV challenge in vaccinated A129. (**a**) Variation in temperatures from 5 days before challenge (dbc) and 10 days post-ZIKV challenge (dac). Lines represent the mean of control (unrelated or mock-control, blue line) and vaccinated groups (red line), (*n* = 6). (**b**) Differences in weight compared to the day of challenge in the control and vaccinated groups. Mouse weight was monitored from day-5 before challenge and up to the date animals were culled, and up to 8 days after challenge. (**c**) Pie charts in percentage that represents the clinical manifestation of the disease (ruffled fur, lethargy, pinched, hunched, wasp-waisted, labored breathing, decrease of mobility and body weight loss), in control and vaccinated groups. (**d**) Kaplan-Meier survival curves with lines representing each vaccinated or control group (*n* = 6). (**e**) ZIKV RNA in mice upon completion of the experiment, day of culling end of the study (21 days after challenge, dac), by RT-PCR. Each dot indicates a mouse from vaccinated or control groups and for each of the brain, ovaries, blood, and spleen tissue. Boxes indicate the mean value and the lines the standard deviation. (**f**) Anti-ZIKV Envelope responses induced by vaccination and measured before challenge.

The ChAdOx1-EDIII vaccine efficacy was additionally assessed in A129 mice undergoing a ZIKV^AF^ heterologous-lineage challenge [35]. Control, mock-vaccinated mice displayed a typical onset of infection after the ZIKV challenge, characterized by an initial increase in body temperature, followed by a drastic decrease (Figure 5a, blue line). In comparison, the ChAdOx1-EDIII vaccine prevented an increase in temperature between days 5–7 (Figure 5a, red line), but displayed the rapid temperature decrease shown in the mock control group. Consistent with the ZIKV challenge model, animals vaccinated with the ChAdOx1 mock control, presented a decrease of body weight (Figure 5b) and clinical symptoms (Figure 5c, blue line), reaching the humane endpoint criteria (80% body weight) between day 7 and 8. In agreement with the previous experimental observations, mice receiving the ChAdOx1-EDIII failed to control the infection and met humane endpoints between day 7 (*n* = 1/6) and day 8 (*n* = 5/6) (Figure 5d, red line). Taken together, we observed coherent results indicating that the ChAdOx1-EDIII vaccine failed to elicit protective immunity in a Type I interferon deficient mouse challenge model.

Measurement of viral load in tissues from euthanized A129 mice indicated that both mock control and ChAdOx1-EDIII vaccinated mice had similar high brain viral loads detectable at day 8 (Figure 5e, Brain). Similar findings were obtained by in-situ hybridization (ISH) in brain tissue (Figure 5f), with the presence of ZIKV in both groups but a trend towards lower levels in the ChAdOx1-EDIII group.

Notably, absence of viral RNA was evident in the ovaries of most ChAdOx1-EDIII vaccinated group (5/6 showed RNA absence), but high levels were detected in the mock control group (Figure 5e, ovaries). Additional ISH in the ovaries demonstrated the presence of ZIKV in the unrelated vaccinated group and a lower but existing burden in the ovaries from the ChAdOx1-EDIII vaccinated group (1 out of 6 mice) (Figure 5f). Similarly, RNAemia at the time of culling (day 7–8) was undetectable in the ChAdOx1-EDIII group and was positive in the mock control groups (Figure 5e, blood). Finally, viral loads in the spleen were similar in both groups with a non-significant trend towards decrease in the ChAdOx1-EDIII group (Figure 5e, spleen). Histological analysis was performed to assess the extent of tissue damage brought about by the ZIKV challenge in both vaccinated groups (Table 1).

## 3. Discussion

Following the ZIKV outbreaks in the Pacific Islands in 2013–2014 and Brazil in 2015, virologists across the world focused their attention and efforts into understanding a pathogen that was relatively overlooked. The urgent need to develop treatments and vaccines prompted the initial strategies to be developed, based on known flaviviruses, particularly DENV.

Although most efforts to develop an effective DENV vaccine used the full E protein (either as a soluble protein or as part of higher structures like VLPs and attenuated/inactivated viruses), there is also a large body of evidence showing that a vaccine based on the EDIII structure could be used as a firm candidate to target flaviviruses. Besides the fact that most anti-EDIII monoclonal antibodies (mAbs) show high viral neutralizing activities, the rationale for investigating EDIII-based vaccines relies mostly on its potential to direct the immune response to a virus-specific, biologically relevant and simple target that is capable of preventing the induction of cross-reacting but poorly-neutralizing anti-FLE antibodies and, thus, reducing the risk of inducing antibody-dependent enhancement (ADE) [38,39,40].

Despite the abundance of EDIII-based vaccine candidates that have were developed for other flaviviruses, to our knowledge the number of similar candidates for ZIKV is scarce and no clinical trials using ZIKV E-DIII were published. Yang et al. produced a vaccine based on ZIKV E-DIII fused with the hepatitis B core antigen (HbcAg-zDIII) to form VLPs in *Nicotiana benthamiana* plants [41], as well as a refolded EDIII from *E. coli* [42]. Immunization with the HbcAg-zDIII VLP vaccine in mice using protocols involving different priming/boost schemes, induced anti-EDIII antibodies and showed evidence of in vitro neutralization, with no induction of ADE against DENV2 [41]. In a similar report, Cabral-Miranda et al. developed a VLP-based approach to display ZIKV E-DIII, using a modified cucumber mosaic virus (CuMVtt-EDIII) [43]; three doses of an adjuvanted CuMVtt-EDIII vaccine induced high titers of anti-EDIII antibodies but low ZIKV neutralizing activities (FRNT50 titer of <1:100). Interestingly, a single-dose and non-adjuvanted administration of CuMVtt-EDIII or EDII alone did not show neutralizing efficacy at 21 days after immunization of the BALB/c mice, but did elicit a high titer of anti-EDIII antibodies, as measured by ELISA. More importantly, given the discrepancies between in vitro and in vivo data, especially regarding protection from viral infection, none of the studies described above included a ZIKV challenge model to test the efficacy of the proposed antigens vaccines.

To further test the suitability of an EDIII-based vaccine to elicit efficacious immunity against ZIKV, we constructed ZIKV-EDIII-based vaccines using three different vaccine platforms—plasmid DNA delivered by a gene gun, protein in adjuvant, and adenoviral-vectored vaccines. DNA gene gun vaccination offers the advantage of a low-cost in vivo delivery of DNA into the skin, allowing the introduction of the genetic material into cells to encode the antigen of interest. Upon production of the protein, cells like skin fibroblasts and dendritic cells initiate a systemic immune response. This design follows our previously reported tetravalent DNA EDIII-based DENV vaccine, which showed that fusion of the EDIII antigen to the dimerizing CH3 domain of the human IgG heavy chain, improved its secretion, thus increasing antigen availability, which boost the immune response when compared to EDIII, which showed lower secretory profiles [28]. As shown by our data, this design also addressed one of the main criticisms attached to genetic vaccines by bringing the immunogenicity of the candidate to levels similar to those measured when using equivalent protein-based antigens. Our previous attempts with the DENV vaccine demonstrated that three 1 μg doses of the EDIII-CH3 DNA vaccine elicited antibodies with high DENV-neutralizing activities in BALB/c mice. In contrast, although the EDIII-CH3 DNA vaccine successfully elicited antibodies against ZIKV E in high titers, which were similar to those induced by an equivalent DENV antigen [28], the virus neutralizing capacity was only modest and notably, none of the tested sera induced 100% neutralization of ZIKV under the conditions tested. Given the disparities between the DENV and ZIKV antigens, we speculated that this could be due to an unexpected compromise regarding antigen secretion or folding in vivo, which could then lead to a limited response against particular epitopes relevant for exerting neutralization.

To test a different strategy for antigen delivery and circumvent any potential limitation poised by the in vivo production of the immunogen, we used the same construct to purify the antigen from mammalian HEK293F cells. In agreement with our DNA-based vaccine, the EDIII-CH3 protein vaccine elicited high anti-EDIII antibody titers and none of the serum reached 100% ZIKV neutralization at the lowest dilution tested. To determine if the neutralizing activities induced by the EDIII-CH3 protein vaccine were sufficient to confer in vivo protection, we challenged vaccinated A129 mice and followed the course of viral infection. Our data showed that even in the presence of high anti-ZIKV E, none of the vaccinated mice were able to successfully control viremia.

To exclude the possibility that the design of the ZIKV EDIII antigen as a CH3-fused dimer could compromise the response to key neutralizing epitopes, we decided to redesign the vaccine as a monomeric EDIII protein using an adenovirus-vectored platform that was previously shown to confer full protection in mice and non-human primates, when using the complete ZIKV E protein as an antigen [36]. In this context, the ChAdOx1 viral-vectored platform was designed to express ZIKV EDIII (ChAdOx1-EDIII) and the vaccine was then tested in two different ZIKV challenge models [35,37]. Besides using an Asian strain instead of an African strain of ZIKV, this antigen differs from the EDIII-CH3 used in the DNA and protein-based vaccines, in the lack of the γCH3 dimerizing domain, which leads to its secretion as a monomeric single-domain peptide, as opposed to a fusion-protein non-covalent dimer. Our results demonstrated that a single and non-adjuvanted immunization using ChAdOx1-EDIII, elicited high anti-ZIKV E antibodies in both BALB/c and A129 mice. Upon ZIKV challenge, a reduction in ZIKV RNAemia was detected in BALB/c mice, but the vaccine was not able to elicit complete protection. Likewise, ChAdOx1-EDIII-vaccinated A129 mice experienced weight loss, temperature-change, and clinical symptoms similar to the mock-vaccinated mice, with detection of virus in the brain and spleen. Of interest, the only two organs where we found a vaccine-induced lower ZIKV load was in ovaries and blood.

Our results consistently showed that all three vaccines readily induced anti-EDIII antibodies, but none was able to completely neutralize ZIKV in vitro and in vivo. Our data indicated that, contrary to DENV, a polyclonal anti-EDIII antibody response at the concentration, specificity, and avidity induced by these three vaccines, only had a limited capacity to neutralize ZIKV. Studies have shown that some anti-ZIKV EDIII mAbs isolated from the ZIKV-exposed patients possess strong neutralizing activities [44,45] and confer protection through passive immunization into IFNAR ^(−/−)^ mice. However, there is evidence that the frequency of human neutralizing antibodies within the overall polyclonal response is lower against DIII than against the fusion loop epitope (FLE) within DII. The latter provides a higher and broader anti-ZIKV neutralization capacity that protect mice against both, African and Asian–American lineages in non-pregnant and pregnant mouse models, preventing maternal–fetal transmission, dam infections, and disease [46]. However, this still remains unknown and might not be sufficient to provide complete protection. Moreover, just as the anti-EDIII response is significantly stronger and more potent in mice than in humans, the nature of the ZIKV antibody response in mice and humans might be significantly different, making it difficult to infer or extrapolate data across species. It should also be considered whether the patients involved in human studies might have had previous DENV infections that shaped the immune response to ZIKV, leading to anti-EDIII antibodies with increased neutralizing strength. Zhao et al. isolated mAbs from mice primed with ZIKV and then boosted with soluble EDIII or ZIKV, with differential binding and neutralization capacities [18]. Of particular interest, mAbs raised in ZIKV-exposed mice and boosted with ZIKV EDIII, could bind monomeric E but they did not bind to ZIKV particles. The more potently neutralizing anti-EDIII mAbs (ZV-67 and ZV-54) recognized the lateral ridge (LR) epitope of EDIII and they were able to bind about 66% of the DIII domains in a mature ZIKV. Passive transfer of both ZV-67 and ZV-54, yielded complete protection upon a ZIKV challenge model in IFN-deficient mice [18]. As the above experiments were performed in a pre-clinical setting, our results in vaccinated mice showing high titers of anti-DIII antibodies but low or poor anti-ZIKV efficacy might be due to an enrichment of antibodies recognizing the EDIII epitopes that are not displayed in infective ZIKV particles. Therefore, it is imperative to design improved antigens that favor a response against relevant neutralizing epitopes in both the therapeutic and the prophylactic settings [47]. Further structurally guided studies are needed to inform the design of relevant immunodominant epitopes to favor the induction of potent neutralizing antibodies. It is important to mention that, in our work, protein conformation and potentially neutralizing epitopes exposure could be different between the DNA-encoded protein, the protein sub-unit or the ChAdOx1 platform with the EDIII presentation on native virions, thus raising the need to perform structural studies using conformation sensitive anti-EDIII antibodies in all expressed proteins. In addition, while correlates or estimates of protection based on the measurement of neutralizing antibodies are established for YFV, WNV, JEV, and TBEV, similar standards of protection are still lacking for DENV and ZIKV and significantly compromise the analysis and extrapolation of data, blurring the lines of what is needed for a vaccine to be considered protective and efficient. Here, we reasoned that in the absence of such standards, the only reliable way to measure the viability of a vaccine candidate was through its ability to either fully neutralize the virus in vitro or provide sterile protection in ZIKV challenge models. Despite neutralizing activities being detected in all cases, none of the three candidates described here comply with this requirement.

To our knowledge, this is the first report that highlight a disparity between the highly immunogenic nature of ZIKV EDIII and the poor neutralizing capacity of the polyclonal antibody response induced against ZIKV EDIII, using different vaccine platforms. More in-depth studies of these responses would significantly increase our knowledge of the effective neutralizing determinants against ZIKV, which could directly inform the development of improved vaccine candidates to achieve protective anti-ZIKV responses, while still avoiding the ADE of DENV.

## 4. Material and Methods

### 4.1. Animals

Female A129 mice were purchased from a home office approved breeder and supplier (B&K Universal Ltd., part of Marshall BioResources, Hull, UK).

For the A129 mice vaccinated with the ChAdOx1 vaccine platform, mice were implanted with a temperature and identity chip upon arrival. During three days’ rest, baseline observations of behavior, temperature, and weight were recorded.

### 4.2. Vaccines

For DNA vaccines, animals were immunized by biolistic delivery of 1 μm gold particles coated with 1 μg of plasmid DNA using Gene Gun technology (Bio-Rad, Hercules, CA, USA), as previously described [28]. Blood samples were collected at day 90 by sub-mandibular puncture and the mice subsequently euthanized using CO2 inhalation. De-complemented pooled sera samples (30 min at 56 °C) were stored at −20 °C until use.

Protein-based vaccine was expressed using the ExpiFectamine™ 293 Transfection Kit (Thermo Fisher Scientific, Abingdon, UK) and purified using the V5-tagged Protein Purification Gel (Caltag Medsystems Ltd., Buckingham, UK) following manufacturer’s instructions.

Adenovirus-based vaccines were prepared in Eppendorf tubes individually prepared for each animal group. Animals were vaccinated in the right hind limb, with 50 μL of vaccine via the intramuscular route at a dose of 10 E^8^IU/mouse.

### 4.3. Animal Ethics

#### 4.3.1. DNA-Based Vaccine

All animal procedures were approved by the ICGEB Animal Welfare Board and the Italian Ministry of Health (Ministero della Salute) (approved protocol DGSAF0024706) and were conducted by adhering to institutional and international guidelines for animal experimentation, and in compliance to laws and policies established in the legislation D. L.vo 26/2014 of the Italian Government.

#### 4.3.2. Protein-Based Vaccine

All animal research related to the protein-based vaccine was approved by the University of Glasgow Animal Welfare and Ethical Board and was carried out under United Kingdom Home Office Licenses, P9722FD8E, in accordance with the approved guidelines and under the UK Home Office Animals (Scientific Procedures) Act 1986 (ASPA).

#### 4.3.3. Adenovirus-Based Vaccine

All animals and procedures were used in accordance with the terms of the UK Home Office Animals Act Project License. Immunization and immunogenicity procedures were approved by the University of Oxford Animal Care and Ethical Review Committee (P9804B4F1).

#### 4.3.4. Pre-Challenge Bleed

One day before challenge, sera was collected from a maximum volume of 100 mL of drawn blood.

#### 4.3.5. Enzyme-Linked Immunosorbent Assay (ELISA) to Quantify Whole IgG

Recombivirus^TM^ Mouse anti-ZIKV envelope protein IgG ELISA kits (Alpha Diagnostic International, RV-403120-1) were used according to the manufacturer’s protocol. In brief, 96-well plates coated with the ZIKV envelope protein were equilibrated with 300 μL of kit working wash buffer. Serial dilutions (3-fold) of sera from vaccinated mice were added. Diluted sera were incubated at RT for 1 h, and after four times washing buffer incubations, 100 μL/well of anti-mouse IgG HRP-conjugate working solution was added for 30 min at room temperature. Plates were washed 5 times and developed for 15 min at room temperature with 100 μL of (TMB) substrate (3,3′,5,5′-tetramethylbenzidine), then stopped by the addition of 100 μL of stop solution. Absorbance was measured at 450 nm on a microplate reader. ELISA ODs were compared between all vaccinated groups at different sera dilutions. For ELISA in BALB/c mice before challenge, antibody endpoint titres are the highest reciprocal serum dilution that resulted in an absorbance >2-fold over the background values, as calculate elsewhere [36]. Anti-Zika envelope antibody concentrations in A129 mice sera before challenge was measured by IgG ELISA using Zika Env antigen as previously described [48].

#### 4.3.6. Challenge Virus

Mice vaccinated with ChAdOx1-based vaccines were challenged using two different lineages. Wild-type mice (Figure 4) were challenged with an intravenous injection of 10^5^ vp (equivalent to 10^2^ PFU) of ZIKV^AS^, consisting of the ZKV2015 (ZIKV BR), as described earlier [36,37]. To extend our observations, the experiment depicted in Figure 5 used A129 mice undergoing a heterologous challenge with a ZIKV^AF^ strain, made with 100 PFU ZIKV^AF^ (MP1751) via the subcutaneous (s.c.) route, to mimic mosquito bite [35]; 40 μL into the right leg and 40 μL into left leg toward the ankle. Mice of the protein vaccine experiment were challenged with 10^4^ PFU Puerto Rican strain ZIKV (PRVABC59) always by the s.c. route, which was performed at a different lab from the ChAdOx1 vaccines.

#### 4.3.7. Clinical Measurements

Over the duration of the study, a daily assessment of temperature and weight was made at least once daily, which included assessment of clinical scores twice a day or more if required during critical periods of the study, to limit animal suffering as a consequence of the infection.

A numerical value was followed for clinical scores—0 normal; 2 ruffled fur; 3 wasp-waisted, pinched, hunched or lethargy; 5 labored breathing, rapid breathing, inactive, neurological; and 10 immobile.

Unnecessary suffering to animals was prevented through the use of humane clinical endpoints by which animals were culled upon reaching any of the following criteria—lack of movement after stimulus-like handling; neurological signs indicated by repetitive movement; weight of approximately 15%–20% from original weight.

#### 4.3.8. Sample Collection

Upon meeting humane clinical endpoints or at the end of the study, mice were culled by the approved methods and blood, spleen, brain, and ovaries were collected at necropsy.

### 4.4. PCR Quantification of Viral Load

#### 4.4.1. Protein-Based Vaccine

Viral RNA was extracted from 10 µL of serum sample, using the QIAamp Viral RNA Kit (Qiagen: Manchester, UK). Real time RT-PCR assay was performed using the One-Step TB Green PrimeScript RT-PCR Kit II (Perfect Real Time) (TaKaRa) (Saint-German-en-Laya, France), with the following amplification conditions—42 °C for 5 min and 95 °C for 10 s, followed by 40 cycles of 95 °C for 5 s and 60 °C for 34 s. The primers used were ZIKV-F: 5′-GTTGTCGCTGCTGAAATGGA-3′ and ZIKV −R: 5′-CGGGACTCTGATTGGCTGTA-3′. A standard curve was generated from ZIKV RNA extracted from 10-fold diluted virus stock with known viral titers in triplicates. The developed real-time RT-PCR assay showed a linear curve with high amplification efficiency and strong correlation (R^2^ = 0.999, Slope—3.31).

#### 4.4.2. Adenovirus-Based Vaccine

Samples were homogenized in PBS using ceramic beads in a Precellys (Lutterworth, UK) automated homogenizer, using a setting of 6 × 5 s cycles of 4500 rpm with a 30 s pause between each cycle. A total of 100 μL of homogenate was transferred to 300 μL RLT buffer (Qiagen, Manchester, UK) for RNA extraction; 25 mL blood were processed using RNAprotect tubes, adding 75 µL of PBS to 300 µL of RLT buffer. After incubation for 10 min, 400 µL of ethanol (70%) were added to the homogenate. The sample was passed through a Qiashredder (Qiagen, Manchester, UK). RNA from ovary and brain was obtained using a Biosprint extraction kit (Qiagen, Manchester, UK) and Kingfisher flex system (ThermoFisher Scientific, Abingdon UK). RNA from spleen and blood was processed using a RNeasy mini kit (Qiagen, Manchester, UK).

A real-time RT-PCR assay for ZIKV was used to quantify viral RNA. Real-time RT-PCR was performed using the SuperScript III Platinum One-step qRT-PCR kit (Life Technologies, Warrington UK). The mastermix (15 μL) consisted of 10 μL of 2x Reaction Mix, 1.2 μL of PCR-grade water, 0.2 μL of 50 mM MgSO4, 1 μL of each primer, ZIKV 1086 (5′-CCGCTGCCCAACACAAG-3′) and ZIKV 1162c (5′-CCACTAACGTTCTTTTGCAGACAT-3′) (REF PMID 18680646), both at 18 μM working concentration, 0.8 μL of probe ZIKV 1107-FAM (5′-AGCCTACCTTGACAAGCAGTCAGACACTCAA-3′) at 25 μM working concentration, and 0.8 μL of SSIII enzyme mix. A total of 5 μL of template RNA was added to the mastermix to give a final reaction volume of 20 μL. Cycling conditions used were 50 °C for 10 min, 95 °C for 2 min, 45 cycles at 95 °C for 10 s with 60 °C for 40 s, and a final cooling step of 40 °C for 30 s. Fluorescence was quantified at the end of each 60 °C step. Analysis was performed using the QuantStudio platform (Applied Biosystems, Warrington UK).

Virus load was quantified in samples using a dilution series of quantified RNA oligonucleotide (Integrated DNA Technologies, Leuven, UK). The oligonucleotide consisted of 77 bases of ZIKV RNA targeted by the assay, using the GenBank accession AY632535.2 synthesized to a scale of 250 nmol through HPLC purification.

#### 4.4.3. Histology

Brain and ovary tissues were fixed in 10% neutral-buffered saline and processed to paraffin wax. Sections of 3–5 μm were stained using hematoxylin and eosin (H&E) to examine by microscope. Lesions were scored subjectively using the following scale—within normal limits, minimal, moderate, and marked. The experiment was blinded to prevent bias.

#### 4.4.4. Contributions

ARS and AHP are the grant holders. ARS directed the project and commissioned the work. CLC designed, constructed, and characterized the adenoviral vaccines. JLSC, MP, and GDL constructed DNA vaccines and subunit EDIII vaccines, respectively. CLC, PA, RAL, JLSC, GDL, and MP, designed and performed the animal experiments. PA and RAL, performed the ZIKV challenge model in the BALB/c mice and the RT-PCR viral loads. CLC, PA, and RAL performed the ELISA assays and analyzed the ZIKV challenge data. SD, VG, and RH conceived and designed the animal experiments in A129 mice. SD, VG, ER, and SFW performed the animal experiments, such as the ZIKV challenge model in A129 mice, the RT-PCR and the histology. MP, GDL, and JLSC performed FRNTs. CLC, JLSC, RAL, and GDL performed in vitro assays and analyzed the ZIKV challenge data. YCK produced ZIKV envelope protein and performed ELISA assays. WD and JM designed, performed, and analyzed in vitro experiments. CLC and MP performed the cell culture, transfections, and the Western blots. AHP, GRS, DHB, ORB, and ASH provided the vital characterized reagents and conceptual support. CLC, GDL, and JLSC analyzed all data. CLC and JLSC wrote the initial draft. All authors read and commented on the manuscript.

## 5. Conclusions

The Domain III (DIII) of the Zika envelope protein is an attractive antigen currently explored as an approach for vaccine development. DIII has given promising results when used as a vaccine for dengue virus and this has prompted its use as a strategy to immunize against the related Zika virus. We investigated the potential of this protein to elicit protective immunity in various mouse models, through the use of three different platforms suitable for vaccine development: plasmid DNA, protein and the chimpanzee adenovirus ChAdOx1. Our results indicate that vaccine-induced immunity to DIII from Zika virus offers limited protection against a challenge with different Zika virus strains comprising the Asian and African lineages. Results were obtained in independent laboratories under different conditions, all yielding similar results of low protective efficacy. We conclude that DIII *per se* is not a promising vaccine candidate and alternative candidates need to be assessed to offer better protection against Zika virus.

## Figures and Tables

**Table 1:** Histology analysis in immunized A129 mice after ZIKV infection.

Vaccine	Animal ID	Histology ID	Culled by Day	Diffusely Scattered Nuclear Debris	Lymphocytic Perivascular Cuffing	Diffusely Scattered PMNs	Degenerating Neurons-Hippocampus	Patchy, Meningeal Infiltration by Inflammatory Cells	Poorly Defined Areas of White Pulp with Large Mononuclear Cells	EMH +/− Apoptosis	Mature PMNs in Red Pulp Sinuses
Vehicle	31,371	732/17	7	Mild	Mild	WNL	WNL	Moderate	Mild	Moderate	Mild
	33,333	733/17	7	Mild	Minimal	Minimal	WNL	Minimal	Mild	Moderate	Mild
	31,110	734/17	7	Minimal	Minimal	WNL	Minimal	Minimal	Mild	Moderate	Minimal
	13,509	735/17	7	Minimal	Minimal	WNL	WNL	Mild	WNL	Moderate	Mild
	31,127	736/17	6	WNL	Minimal	WNL	WNL	Minimal	WNL	Moderate	Mild
	13,035	737/17	7	Moderate	Moderate	Minimal	Moderate	Moderate	Mild	Moderate	Minimal
ChAdOx1 EDIII	31,764	714/17	8	Moderate	Moderate	WNL	Marked	Moderate	WNL	Moderate	WNL
	12,300	715/17	8	Mild	Mild	WNL	Moderate	Moderate	WNL	Moderate	Minimal
	31,303	716/17	8	Mild	Moderate	WNL	Minimal	Moderate	Minimal	Moderate	Mild
	31,131	717/17	8	Mild	Mild	Minimal	WNL	Moderate	Minimal	Moderate	Mild
	13,219	718/17	7	Minimal	Moderate	WNL	Minimal	Moderate	WNL	Moderate	Minimal
	31,398	719/17	8	Minimal	Moderate	WNL	Minimal	Moderate	Minimal	*Severe*	Mild
Reference	13,657	690/17	21	WNL	WNL	WNL	WNL	WNL	WNL	Minimal	WNL
	12,304	691/17	21	WNL	WNL	WNL	WNL	WNL	WNL	Mild	WNL
	13,545	692/17	21	WNL	WNL	WNL	WNL	WNL	WNL	Mild	WNL
	31,609	693/17	21	WNL	WNL	WNL	WNL	WNL	WNL	Mild	WNL
	15,214	694/17	21	WNL	WNL	WNL	WNL	Minimal	WNL	Mild	WNL
	13,448	695/17	21	WNL	WNL	WNL	WNL	WNL	WNL	Minimal	WNL
ChAdOx1 Mock	31,220	726/17	8	Minimal	Moderate	WNL	Mild	Mild	Mild	Moderate	Mild
	13,690	727/17	8	Mild	Moderate	WNL	Mild	Moderate	Minimal	Moderate	Moderate
	34,144	728/17	8	Minimal	Mild	Mild	Moderate	Moderate	Minimal	Severe	Mild
	13,122	729/17	8	WNL	Mild	WNL	Not present	Minimal	Not present		
	12,189	730/17	7	Minimal	Mild	WNL	WNL	Moderate	Minimal	Moderate	Mild
	13,555	731/17	7	Minimal	Minimal	Minimal	WNL	Mild	Mild	WNL	Mild
Vehicle	31,371	732/17	7	Mild	Mild	WNL	WNL	Moderate	Mild	Moderate	Mild
	33,333	733/17	7	Mild	Minimal	Minimal	WNL	Minimal	Mild	Moderate	Mild
	31,110	734/17	7	Minimal	Minimal	WNL	Minimal	Minimal	Mild	Moderate	Minimal
	13,509	735/17	7	Minimal	Minimal	WNL	WNL	Mild	WNL	Moderate	Mild
	31,127	736/17	6	WNL	Minimal	WNL	WNL	Minimal	WNL	Moderate	Mild
	13,035	737/17	7	Moderate	Moderate	Minimal	Moderate	Moderate	Mild	Moderate	Minimal
ChAdOx1 EDIII	31,764	714/17	8	Moderate	Moderate	WNL	Marked	Moderate	WNL	Moderate	WNL
	12,300	715/17	8	Mild	Mild	WNL	Moderate	Moderate	WNL	Moderate	Minimal
	31,303	716/17	8	Mild	Moderate	WNL	Minimal	Moderate	Minimal	Moderate	Mild
	31,131	717/17	8	Mild	Mild	Minimal	WNL	Moderate	Minimal	Moderate	Mild
	13,219	718/17	7	Minimal	Moderate	WNL	Minimal	Moderate	WNL	Moderate	Minimal
	31,398	719/17	8	Minimal	Moderate	WNL	Minimal	Moderate	Minimal	*Severe*	Mild
Reference	13,657	690/17	21	WNL	WNL	WNL	WNL	WNL	WNL	Minimal	WNL
	12,304	691/17	21	WNL	WNL	WNL	WNL	WNL	WNL	Mild	WNL
	13,545	692/17	21	WNL	WNL	WNL	WNL	WNL	WNL	Mild	WNL
	31,609	693/17	21	WNL	WNL	WNL	WNL	WNL	WNL	Mild	WNL
	15,214	694/17	21	WNL	WNL	WNL	WNL	Minimal	WNL	Mild	WNL
	13,448	695/17	21	WNL	WNL	WNL	WNL	WNL	WNL	Minimal	WNL
ChAdOx1 Mock	31,220	726/17	8	Minimal	Moderate	WNL	Mild	Mild	Mild	Moderate	Mild
	13,690	727/17	8	Mild	Moderate	WNL	Mild	Moderate	Minimal	Moderate	Moderate
	34,144	728/17	8	Minimal	Mild	Mild	Moderate	Moderate	Minimal	Severe	Mild
	13,122	729/17	8	WNL	Mild	WNL	Not present	Minimal	Not present		
	12,189	730/17	7	Minimal	Mild	WNL	WNL	Moderate	Minimal	Moderate	Mild
	13,555	731/17	7	Minimal	Minimal	Minimal	WNL	Mild	Mild	WNL	Mild

Histological lesions were assessed in ChAdOx1-EDIII vaccinated mice and control groups. As a comparison, mice were vaccinated with a reference vaccine consisting of a ChAdOx1 prME ΔTM, which provided 100% protection against the ZIKV challenge. The table shows the day of culling for each mice. For the brain and spleen, 5 and 3 different microscopic measurement observations were performed, respectively. Scores are within normal limits (WNL) (dark green), minimal (light green), mild (yellow), moderate (dark red), and severe (light red).
